# Factors influencing contraceptive use or non-use among Aboriginal and Torres Strait Islander people: a systematic review and narrative synthesis

**DOI:** 10.1186/s12978-020-01004-8

**Published:** 2020-10-15

**Authors:** Jacqueline Coombe, Amy E. Anderson, Natalie Townsend, Kym M. Rae, Stephanie Gilbert, Lyniece Keogh, Christine Corby, Deborah Loxton

**Affiliations:** 1 Melbourne School of Population and Global Health, The University of Melbourne, Level 3, 207 Bouverie Street, Carlton, VIC 3053 Australia; 2 Research Centre for Generational Health and Ageing, Hunter Medical Research Institute, The University of Newcastle, Level 4 West, University Drive, Callaghan, NSW 2308 Australia; 3 Mater Research Institute, Level 3, Aubigny Place, Raymond Terrace, South Brisbane, Qld 4101 Australia; 4 Aboriginal and Torres Strait Islander Studies Unit, The University of Queensland, Staffhouse Road, St Lucia Campus, Brisbane, QLD 4072 Australia; 5 Tamworth Local Aboriginal Land Council, 2/1 Hinkler Street, Tamworth, NSW 2340 Australia; 6 Walgett Aboriginal Medical Service, 37 Pitt Street, (PO Box 396), Walgett, NSW 2832 Australia

**Keywords:** Systematic review, Contraception, Contraceptive use, Contraceptive services, Contraceptives, Aboriginal, Torres Strait Islander, Indigenous, Australia

## Abstract

**Background:**

The Australian population has an unmet need for contraception. However, evidence suggests contraceptive patterns of Aboriginal and Torres Strait Islander populations are unique. To tailor contraceptive services and meet the contraceptive needs of Aboriginal and Torres Strait Islander people, it is important to understand the contributing factors to contraceptive use and non-use.

**Methods:**

This study aimed to systematically review and narratively synthesise the evidence exploring the factors influencing contraceptive use among Aboriginal and Torres Strait Islander people. A systematic literature search was initially run in September 2016 and was updated again in April and August of 2018. A qualitative narrative synthesis was conducted from 2018 to 2019. Factors influencing contraceptive use or non-use were explored using a Social Ecological Model.

**Results:**

The review identified 17 studies meeting the inclusion criteria published between 1972 and 2018. Most of the included studies were qualitative (*n* = 11), with the remaining studies being mixed methods (*n* = 3) or quantitative (n = 3). The majority focused on either a localised geographic area or specific Aboriginal or Torres Strait Islander community (*n* = 11). One study specifically focused on factors influencing contraceptive use, albeit among postpartum women. The remaining studies discussed factors influencing contraceptive use within the context of risky behaviour, sexual transmitted infections, or contraceptive practices more generally. Factors unique to individual communities included community attitudes (e.g. importance of not being too young to have a baby), specific cultural norms (e.g. subincising the penis as part of transition to manhood), and access to culturally appropriate health services. Other factors, including contraceptive characteristics (e.g. discomfort of condoms) and reproductive coercion (e.g. partner wants a baby), were similar to those found in the broader population of Australia and internationally. Most studies were lacking in quality, warranting more methodologically sound studies in the future to further assess the factors contributing to contraceptive use or non-use among Aboriginal and Torres Strait Islander people.

**Conclusions:**

Identifying community specific facilitators, as well as understanding the more broadly applicable factors contributing to contraceptive use or non-use, is essential if wanting to offer appropriate contraceptive services within an Aboriginal or Torres Strait Islander community.

## Plain English summary

The Australian population has an unmet need for contraception. However, evidence suggests contraceptive patterns of Aboriginal and Torres Strait Islander populations are unique. To tailor contraceptive services and meet the contraceptive needs of Aboriginal and Torres Strait Islander people, it is important to understand the contributing factors to contraceptive use and non-use. This study aimed to systematically review and narratively synthesise the evidence exploring the factors influencing contraceptive use among Aboriginal and Torres Strait Islander people. The review identified 17 relevant studies. The majority focused on either a localised geographic area or specific Aboriginal or Torres Strait Islander community. One study specifically focused on factors influencing contraceptive use, albeit among postpartum women. The remaining studies discussed factors influencing contraceptive use within the context of risky behaviour, sexual transmitted infections, or contraceptive practices more generally. Factors unique to individual communities included community attitudes, specific cultural norms, and access to culturally appropriate health services. Other factors were similar to those found in the broader population of Australia and internationally. Identifying community specific facilitators, as well as understanding the more broadly applicable factors contributing to contraceptive use or non-use, is essential if wanting to offer appropriate contraceptive services within an Aboriginal or Torres Strait Islander community.

## Background

According to data from the Second Australian Study of Health and Relationships completed in 2012–13, women in Australia have an unmet need for contraception (defined as at risk of pregnancy, but not using contraception), even with access to a wide range of options, spanning barrier, hormonal, long-acting and permanent methods^1^ [1]. In this representative sample of 5654 Australian women, the oral contraceptive pill, condoms and sterilisation were most popular, while long-acting reversible contraception (LARC; contraceptive implant, intrauterine device) were less popular [1]. Contraceptive practices at a population level in Australia are relatively clear, with most women of reproductive age using the oral contraceptive pill and condoms, with the use of these methods declining as women age and reproduce [1]. However, there has been comparatively little research examining the contraceptive practices of marginalised groups, particularly Aboriginal and Torres Strait Islander peoples. For the purposes of this research, Aboriginal and Torres Strait Islander peoples will respectively be used to describe the hundreds of communities with diverse cultures that make up the First Nations people of mainland Australia and the islands of the Torres Strait. The current research was developed after a 3 year consultation period with Aboriginal community members who identified reproductive health as an area of need as discussed in more detail in the Cultural Guidance section below.

The current evidence base suggests that patterns of contraceptive use differ between Aboriginal and Torres Strait Islander peoples and non-Indigenous Australians [2]. This is not surprising considering the post-colonisation history of contraceptive use enforced upon Aboriginal and Torres Strait Islander women by the predominantly non-Indigenous Australian population which has included: i) the use of Depo-Provera without fully informed consent as a form of short term infertility, ii) sterilisation for unexplained reasons, iii) prevention of reproduction through forced removal of children, and iv) criminalisation of having children with other members of Aboriginal and Torres Strait Islander communities [3]. It is unknown how this history of forced contraception and removal of children may have influenced current attitudes towards contraceptive use or non-use. The contraceptive needs of Aboriginal and Torres Strait Islander communities may vary from those of non-Indigenous Australians due to varied lived experiences as a result of colonisation. Therefore, it is important to identify any specific unmet needs for this population to ensure contraceptive services are tailored to be culturally appropriate, safe and acceptable for each Aboriginal and Torres Strait Islander community.

There are limited data on the contraceptive use of Aboriginal and Torres Strait Islander peoples. Data collected in the 2012–13 National Aboriginal and Torres Strait Islander Health Survey suggests less than half of participants were using contraception at the time of the survey (49%; the Australian national average was 67% in 2015) [2]. While the wording of the survey questions regarding contraceptive use limits comparability of findings, data reported here suggests the oral contraceptive pill followed by the contraceptive implant were the most popular methods used [2]. These findings contrast with those reported elsewhere. A 2007 study using data from The Household Income and Labour Dynamics in Australia (HILDA) Survey found that 71 Aboriginal and Torres Strait Islander women aged 18–44 years reported using the contraceptive injection more than other Australian women (*n* = 2150), who were more likely to use other forms of contraception [4]. However, the data is now more than 10 years old and may no longer accurately reflect patterns of contraceptive use. In a study which examined the contraceptive practices of women (aged 12–50 years) in three remote Aboriginal communities in Western Australia, high rates of LARC use were reported among the 121 women currently using a prescribed contraception in 2014, with 77% of the sample using the contraceptive implant, and 7% the contraceptive injection [5]. In a further study using data from a cross-sectional national survey, women reporting intrauterine device (IUD) use were 11 times more likely to identify as Aboriginal and/or Torres Strait Islander, compared to non-users, although these findings should be interpreted with caution given the very small number (*n* = 29) of Aboriginal and Torres Strait Islander respondents [6].

As has been noted elsewhere [5], there is a persistent research focus on sexually transmitted infections (STI) transmission and prevention in Aboriginal and Torres Strait Islander communities, with little exploration into contraceptive practices more generally. Although STI’s are an important focus and an essential component of providing good sexual and reproductive healthcare, understanding contraceptive practices in the context of family planning more generally, as well as the factors contributing to use or non-use are just as vital. This review aimed to examine the factors influencing contraceptive use among Aboriginal and Torres Strait Islander peoples. Where possible, gender specific factors were identified.

## Methods

A systematic review, following the Preferred Reporting Items for Systematic Reviews and Meta-Analyses (PRISMA) guidelines [7] was conducted to meet the aims of this study. This review was registered with PROSPERO (record number: CRD42018094434, https://www.crd.york.ac.uk/prospero/display_record.php?RecordID=94434).

### Eligibility criteria

Only peer-reviewed studies involving Aboriginal or Torres Strait Islander peoples living in Australia reporting on primary data, including factors contributing to contraceptive use or non-use, were included. Studies were excluded if they did not include Aboriginal and Torres Strait Islander peoples, or did not include Aboriginal and Torres Strait Islander peoples as a sub-group where data could be extracted separately from non-Indigenous participants, or did not include primary data. No restrictions on date of publication, study sample size or methodology were imposed.

### Information sources

Medline, Embase, PsycInfo, Cochrane Library, CINAHL, Informit and Scopus were searched in September 2016, and the search was updated in April 2018 using the original search terms. A final check of the databases was conducted in August 2018 prior to finalisation of the narrative synthesis (i.e. 2018–2019).

### Search strategy

The search terms included three main categories: 1) country (e.g. search terms: Australia; austral*), 2) population group (e.g. search terms: Oceanic Ancestry Group; First Australian; aborig* or indigen* or torres strait*; Indigenous people) and 3) contraception (e.g. search terms: Contraception Behaviour; Contraceptive Agents; birth control; family planning). Search terms were entered according to the requirements of each database (e.g. MeSH terms for Medline). Searches were limited to human studies. A full list of search terms are listed in the registered protocol (https://www.crd.york.ac.uk/prospero/display_record.php?RecordID=94434).

### Study selection

All articles were downloaded into Endnote, and duplicates removed. Titles and abstracts were independently screened by two reviewers (JC and NT) and sorted accordingly. Articles deemed eligible for inclusion, articles where it was difficult to discern eligibility from the title and abstract (i.e. ‘unsure’ articles), and articles where the reviewers disagreed upon eligibility progressed to the next phase of screening. Articles were excluded at this phase with reasons for this exclusion recorded (including, no Aboriginal or Torres Strait Islander focus, or no contraception focus). Full-texts were then retrieved, and screened independently for eligibility by two reviewers (JC and NT). The reviewers (JC and NT) compared their screening, and discussed any disagreements, before agreeing on the final articles to be included in the review. Further details regarding study selection, including the number of studies removed at each stage are available in Fig. 2.

### Data items

Data extracted for each study included: citation; study design; study population; participant demographics and baseline characteristics; recruitment and study completion rates; outcomes and times of measurement; information for quality assessment (based on the study design); and reported factors contributing to contraceptive use or non-use (noting any instances where these were gender specific). Data extraction was undertaken by one reviewer (JC).

### Quality assessment

Quality assessment was conducted for all included studies. Quantitative studies were assessed using the Quality Assessment Tool for Observational Cohort and Cross-Sectional Studies [8], qualitative studies were assessed using the Critical Appraisal Skills Program (CASP) Qualitative Research Checklist [9], and a scoring system for appraising mixed methods research was used to assess mixed methods studies [10].

### Narrative synthesis

A narrative synthesis approach was utilised to explore the factors contributing to contraceptive use or non-use reported in the included studies. NVivo software was used to manage data during coding. The synthesis was conducted using three levels of coding. For the first level of coding, one author (JC) coded the text in the results sections of each article. This coding was similar to the generation of initial codes proposed by Braun and Clarke [11], and the line-by-line coding suggested by Thomas [12], and kept as close to the findings reported in each study as possible. Qualitative, quantitative and mixed methods studies were all coded, with the numerical data and its accompanying textual explanation coded. Numerical data presented in tables was not coded. Coding was iterative, where new codes were generated as needed, and existing codes used where appropriate. Once all articles were coded, codes were organised into descriptive themes, a process akin to the searching and reviewing themes stage utilised by Braun and Clarke [11], and the descriptive coding used by Thomas [12]. These codes were broadly categorised as either factors contributing to contraceptive use or non-use. Codes that were specifically focused on gendered issues were grouped together (for example, perceptions regarding differing roles for men and women in using condoms). In line with the recommendations of Thomas [12], and similar to the defining and naming themes step utilised by Braun and Clarke [11], analytical themes were developed, which specifically addressed the aim.

**Fig. 1:**
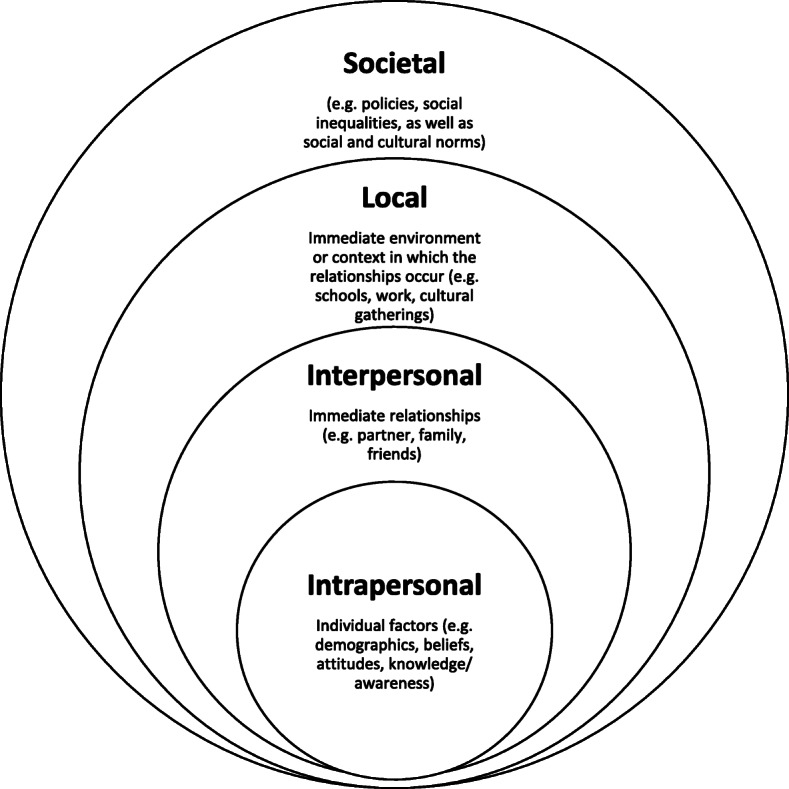
Social Ecological Model adapted from Coleman and Alonso 2016 [15]

The second level of coding involved the application of a Social Ecological Model by a second coder (AA), to assist in explaining and understanding the findings. Ecological frameworks typically incorporate multi-level factors to explain complex phenomenon [13], which made this framework particularly useful in this context. The Violence Prevention Alliance of the World Health Organization has provided an ecological framework for the prevention of violence, consisting of an individual, relationship, community and societal level [14]. This model has been adapted and applied to a number of topics, including family planning and contraceptive use [15, 16]. For this narrative synthesis, four levels adapted from Coleman and Alonso’s (2016) Social Ecological Model [15] were utilised, as seen in Fig. 1. As the word community has a different meaning within Aboriginal and Torres Strait Islander cultures, it was deemed more appropriate to rename the Community level of Coleman and Alonso’s (2016) model to Local level [15].

The third level of coding consisted of an arbitration process between the two coders (JC and AA) and the senior author (DL). The coding applied based on the Social Ecological Model was discussed until consensus was met by all three parties.

### Cultural guidance

To ensure this research was conducted in a culturally appropriate and safe manner, the research team engaged in a consultation process over approximately a 3 year period. The aim of this process was to establish strong relationships with appropriate Aboriginal community members to assist with identifying any areas of need during the conceptualisation of this research. The research team developed relationships with two Chief Executive Officers of Aboriginal Medical Services (one based in a rural community and one based in a remote community), Aboriginal researchers, and Elders. Informal discussions with these community members led to the identification of reproductive health as an area of need.

The review should be interpreted with caution, as the literature search and narrative synthesis were conducted by non-Aboriginal authors. However, steps were taken to ensure the review was conducted in a culturally safe and appropriate manner. Specifically, cultural guidance and feedback was sought from members of Aboriginal communities when drafting the manuscript to ensure sensitivity in language and interpretation of results, including coding. Authorship was warranted based on the invaluable contribution of cultural advice. Final approval was gained before submitting the manuscript for publication.

## Results

**Fig. 2:**
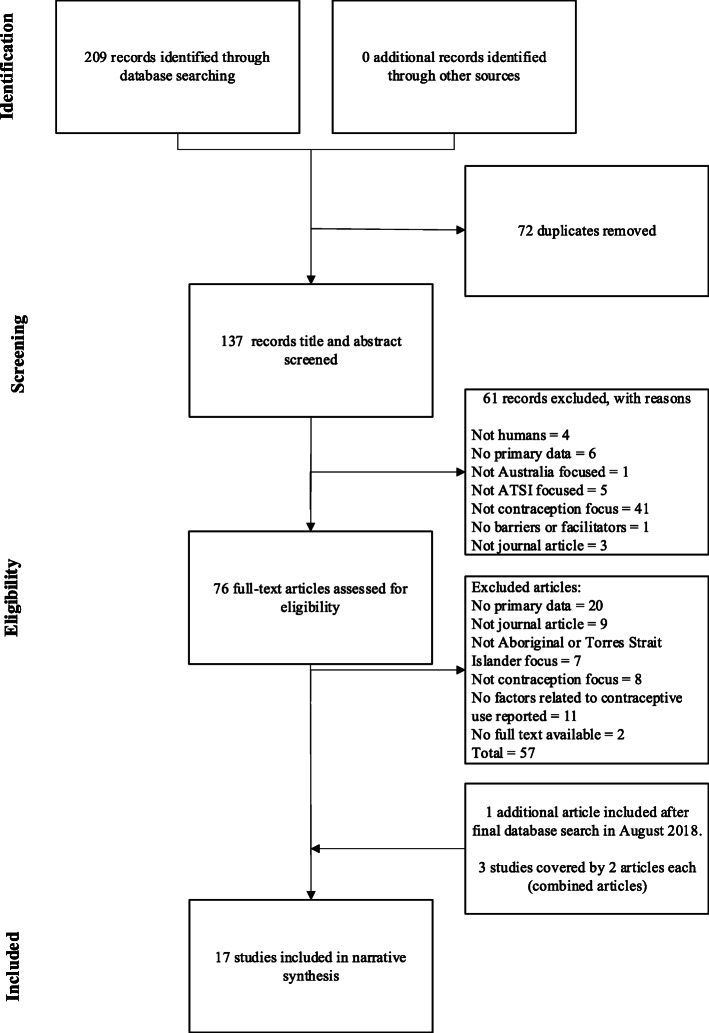
PRISMA diagram detailing the search and selection process for included studies

In all, 209 records were identified via our database screening, of which 20 articles were deemed relevant to this review. Three authors published two papers each from their respective studies [17–22], so the final number of included studies was 17. See the PRISMA diagram [7] in Fig. 2 for an overview of this process.

### Characteristics of included studies

The majority of studies were qualitative (11/17), followed by mixed methods (3/17) and quantitative (3/17). Six studies were conducted in Queensland (QLD), five in the Northern Territory (NT), two in Western Australia (WA), one each in New South Wales (NSW) and South Australia (SA) and two across multiple states (NSW, WA, SA, QLD and NT, WA, SA). Eleven studies were conducted in a localised geographic area or with specific Aboriginal and Torres Strait Islander communities. It was unclear how many studies included Torres Strait Islander participants, as three studies described their study populations as Indigenous or as Aboriginal and Torres Strait Islander only, with no further clarification [23–25]. Only one study reported the specific inclusion of Torres Strait Islander participants, although results were pooled for all Indigenous participants [17, 18]. Two studies reported data from both Indigenous and non-Indigenous participants [23, 26], one of which did not disaggregate findings based on participant Indigenous status [26]. Four studies reported data collection occurring from 2010 or later, six started data collection prior to 2010, and seven studies did not report or were unclear about when data collection occurred.

The majority of included studies focused on women only (9/17) or on women and men (7/17), and only one study focused on men. Nine studies focused on ‘young’ people, aged below 35 years, while the remainder focused on participants aged across entire lifespans, or did not report the ages of their participants. Eight studies examined contraceptive or sexual health practices or behaviours more generally, while eight specifically explored these practices in relation to STI transmission and risky sexual behaviour. One study specifically examined factors influencing contraceptive use, although this study focused on the post-partum period. All included studies, with their key findings and limitations, are summarised in Table 1.

**Table 1:** Characteristics of included studies

First Author	Aim	Design	Location	Participants	Key Findings & Limitations
**Bryant 2011** [27]	Explore condom use in the context of sexual risk behaviour and STI transmission	Quant (survey)	NSW (34% from regional, remote or rural areas)	293 Aboriginal men and women, aged 16–30 years	• Majority of participants were sexually active but condom use was inconsistent, intermittent or non-existent • Potential bias due to non-probability sampling, public recruitment and self-reported data
**Cox 1972** [28]	Explore contraceptive practices, and acceptability of contraception	Qual (NR^a^)	SA (one remote community)	108 Pitjantjatjara women	• Overview of contraceptive practices and attitudes in community, and recommendations for culturally appropriate care • Study methodology and participant demographics not reported
**Gray 1987** [29]	Explore the family planning practices among women, in the context of (reported) fertility decline among Aboriginal people in the 1970’s	Qual (NR)	NSW, SA, WA, QLD (five communities)	251 Aboriginal women, aged 15–50 years	• Most women were aware of the contraceptive methods available to them • Over half of the women approved of contraceptive use in some circumstances, such as to space apart children • Clear differences of opinion seen between five distinct communities • Substantial number of women claimed no opinion in relation to one of the specific or general uses of family planning, highlighting sensitivity of the topic area • Little information regarding the methodology employed and participant demographics not adequately reported
**Griffiths 2016** [5]	Assess the use, effectiveness and acceptance of prescribed contraception in three communities (focus on LARC)	Mixed (Retrospective file review, semi-structured interviews)	WA (three remote communities)	Health records of 191 Aboriginal women, aged 12–50 years 20 additional women were interviewed	• High rates of LARC uptake, continuation rates comparable to those reported elsewhere, suggesting the acceptability of these methods. • Contraceptive use potentially under-reported in these communities • Women not using contraception were not represented
**Helmer 2015** [23]	Examine sexual behaviour and decision making in the context of everyday life experience and aspirations of Indigenous and non-Indigenous Australians	Qual (Group discussions, body mapping, interviews)	NT, WA, SA (urban and rural sites)	171 total participants, 88 of which identified as Indigenous, aged 16–25 years	• Sex education provided in schools did not meet the needs of young people studied • Findings limited to the context of sex education • Condoms were the only form of contraception discussed in the paper
**Ireland 2015** [30]	Explore and describe young women’s behaviour and knowledge in relation to sexual health	Qual (Ethnography)	NT (one remote community)	12 Aboriginal women aged 16–33 and 19 Aboriginal women aged 40–90	• Lack of sexual health knowledge and risky sexual behaviours reported • Women dissatisfied with the physical consequences of their contraceptive method were unaware of alternative choices • Lack of generalisability to the broader population (little participant demographic information reported)
**James 2018** [31]	Examine the factors influencing postpartum contraception	Qual (semi-structured interviews, focus groups)	QLD (one urban Community-Controlled Health Organisation)	17 Aboriginal women aged ≥16 years, who were less than 12 months post-partum	• Most participants reported a desire for postpartum contraception, but reported barriers to accessing and using their preferred methods • Sample did not include Torres Strait Islander women and had limited representation of women from remote areas
**Johnston 2015** [26]	Describe the views of sexual health service providers on access issues for young people and consider them with the views of young people themselves	Mixed (semi-structured interviews, survey)	QLD (four towns, regional and rural)	32 service providers (2 Aboriginal health workers) and 391 young people aged 15–24 years (11.3% Aboriginal and/or Torres Strait Islander)	• Attitudes of service providers and their relationship with youth are more significant to young people than currently perceived by service providers themselves • Only briefly reported on factors influencing contraceptive use for Aboriginal and Torres Strait Islander youth. Sampling strategy purposive, may be some selection bias
**Larkins 2007, 2011** [17, 18]	To explore the attitudes to pregnancy and parenthood among a group of Indigenous young people To gain an understanding of the attitudes and behaviours of Indigenous young people regarding relationships, contraception and safe sex	Mixed (Survey, focus groups)	QLD (Townsville)	186 Indigenous people aged 12–18 years, and 10 Indigenous women with children or pregnant	• Many held idealised notions of parenthood • Motherhood was considered transformative, and an opportunity to make positive lifestyle changes for the sake of the baby • Small number of interview participants (*N* = 10) limits generalisability, limited reporting of survey (*N* = 186) and focus group results (*N* = 59) • Nearly half of participants were sexually active • In survey responses, 60% of participants reported condom use, and 26% reported hormonal contraceptive use • Barriers to use were reported • Self-reported data, sample not representative or generalisable
**Mooney-Somers 2012** [24]	Examine how young Indigenous Australians keep themselves healthy and protected against STIs	Qual (Interviews)	QLD (Townsville)	45 men and women aged 17–26 years who self-identified as Aboriginal and Torres Strait Islander, at risk of or experiencing homelessness	• Health behaviours are complex, and not static over time • Condom use contingent on sexual partner, relationship, context and access • Focus of the paper is homelessness, and findings should be interpreted within this context
**Reid 1979a, 1979b** [19, 20]	Review the attitudes of Aboriginal women towards family size, spacing and planning, and explore attitudes towards childbearing and family planning	Qual (Interviews)	Northern Australian community	92 Aboriginal women, aged ≥15 years	• Lack of culturally appropriate services in community • Many participants had positive attitudes towards contraception, and reported preferences for family size and spacing • Little methodological information provided
**Roberts 1997** [32]	Investigate the attitudes of Aboriginal women towards the use of condoms to prevent HIV and other STIs	Qual (Interviews)	NT (Darwin)	12 Aboriginal women, aged 19–44	• Although participants were aware of condoms and their protection against STI’s, few used them, and they were generally considered unfavourably • Small study of limited generalisability. All participants were students in university preparation courses
**Samisoni 1980a, 1980b** [21, 22]	Explore family planning and contraceptive practices among Aboriginal women	Qual (Semi-structured interviews)	QLD (Brisbane)	236 Aboriginal women	• Oral contraceptives were the most popular method used, although many reported unintended pregnancies in the context of contraceptive use • Experiences of side effects impacted continuation rates • Little methodological information provided and participant demographic information lacking
**Scott 2015** [25]	Explore sexual risk and healthcare seeking behaviour among Aboriginal and Torres Strait Islander youth	Quant (Survey)	QLD (Townsville)	155 Aboriginal and Torres Strait Islander people, aged 16–24 years	• Three quarters of participants reported carrying condoms at least sometimes, and 82% had used a condom in their last casual sexual encounter • Men were more likely to report condom use than women. • Non-random selection of sample not generalisable to broader population • Peer interviewers known to participants, which may have impacted responses to the interviewer-administered survey • Data self-reported, which may be subject to recall bias
**Stark 2007** [33]	Examine current levels of knowledge regarding STIs and their transmission, perception of risk of STIs, patterns of, access to and experiences with negotiating condom use	Qual (Interview)	NT (one remote community)	24 Aboriginal women, aged 18–35 years	• Poor knowledge of STI transmission, limited condom access and limited condom use was reported • Sexual activity in the context of alcohol use, reduced ability and/or desire to negotiate condom use • Small sample limits generalisability. • Participant responses may have been impacted by relationship with the researcher (a non-Aboriginal woman and nurse in the community), cultural and linguistic misunderstandings in questions and answers, and sensitive nature of the face-to-face interviews
**Williams 2015** [34]	Describe the sexual health behaviour, alcohol and other drug use and health service use among young people	Quant (Survey)	WA (Perth, and south-west WA)	244 Aboriginal men and women, aged 16–30 years	• Participants initiated sexual activity at a young age • Men reported carrying condoms more often than women, and men also reported use at last casual sex more often than women • Data should be interpreted cautiously, as there were high non-response rates to questions about sexual behaviours
**Willis 2003** [35]	Report on the culture-specific barriers that masculinity poses to preventing HIV transmission among Pitjantjatjara men.	Qual (Ethnography)	NT (remote communities)	Pitjantjatjara and Yankunytjatjara men	• Significant cultural barriers to condom use were reported • Little methodological information provided, and participant demographics lacking

^a^*NR* not reported

### Quality assessment

Eleven qualitative studies were assessed. In all studies but one [28], the qualitative methodology was deemed appropriate. Cox and Burden was not considered further as there was insufficient information to make an assessment [28]. Only two studies [30, 31] included all items in the CASP Qualitative Research Checklist [9]. Four studies included all but one item [5, 24, 32, 33]; missing items included a reflexivity statement, ethical statement or rigorous data analysis. The remainder of the studies either had insufficient, or no information for assessment of specific criteria. Sufficient information regarding recruitment, data collection, researcher reflexivity, ethical issues, and information regarding data analysis were often lacking in these studies.

Three studies using quantitative methods were assessed, and all were cross-sectional. All three studies either did not report items, or did not provide sufficient information for assessment. Of the 12 items on the Quality Assessment Tool for Observational Cohort and Cross-Sectional Studies [8], the majority of the quality criteria items were either not met or not reported by the included studies. For example, participation rates were not reported as convenience samples were used in all three studies, increasing risk of selection bias.

Four studies were assessed as mixed methods using the scoring system for appraising mixed methods research [10]. All four studies reported on qualitative checklist items, except researcher reflexivity, which was not reported in any study. Information provided for the quantitative aspects of the studies varied. None of the studies reported controlling for confounding variables, and only two studies justified the measurements chosen and the mixed methods design [5, 26]. However, all studies integrated qualitative and quantitative results.

Overall, there is a lack of high quality evidence in this area. Although some of the qualitative work was deemed appropriate, most was lacking in high quality research methods and reporting, particularly in regards to ethics statements and rigorous data analysis. There is a lack of quality evidence from the quantitative and mixed methods research conducted to-date.

### Factors influencing contraceptive use

**Fig. 3:**
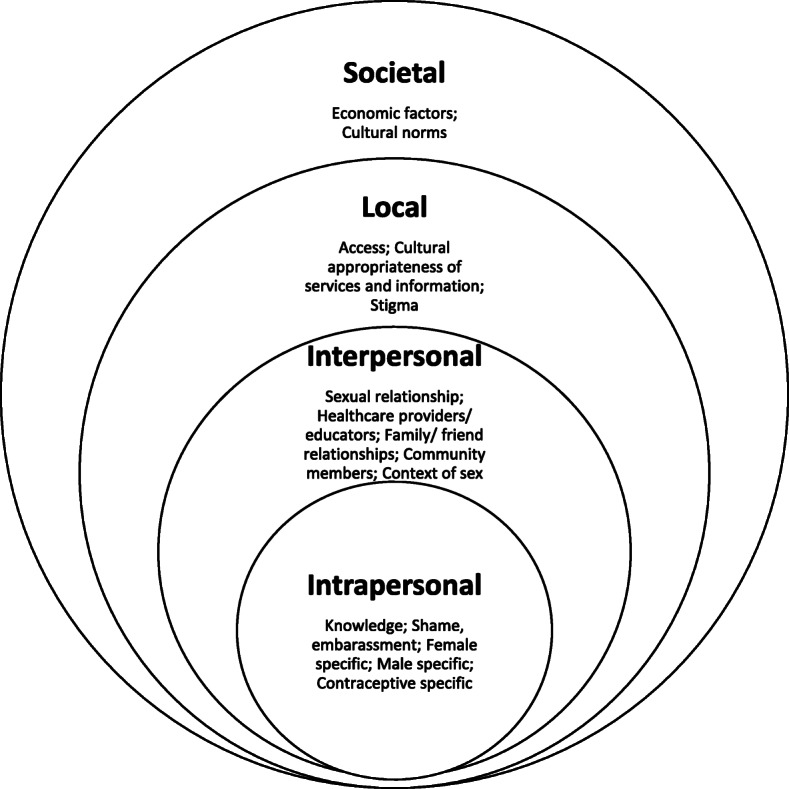
Social Ecological Model of key factors influencing contraceptive use and non-use

A multitude of factors that influenced use and non-use of contraception among Aboriginal and Torres Strait Islander peoples were reported, with reasons for non-use being more prevalent in study reporting. Each Aboriginal community across Australia is unique, however, they share a history of forced contraception. Although requiring further investigation as to how it relates to current attitudes towards contraceptive use, such a history may have contributed to the number of common factors found by this review, which were reported as influencing contraceptive use and non-use. Although these factors were identified as common across communities, the individuality of each community is also acknowledged. Table 2 includes a summary of each of the key factors influencing contraceptive use and non-use arising from the narrative synthesis, organised within a Social Ecological Model. As the levels of the model are embedded within each other, so were the many factors influencing use and non-use of contraception as visualised in Fig. 3. The factors at all levels were intertwined and were clearly influenced by factors at other levels.

**Table 2:** Factors influencing use and non-use of contraception

Social Ecological Model Level	Theme	Factors influencing non-use	Factors influencing use
Intrapersonal	Knowledge	• Misperceptions/misinformation about implant [5] • Lack of knowledge about available methods [5, 19–22, 29–31] • Lack of knowledge about sexual and reproductive health generally [18] • Information about contraception from non-medical sources [21, 22] • Do not know where to access condoms in community, how much they cost, or know where but not how [32, 33] • Overwhelming information on internet, difficult to identify reliable information [31]	• Able to identify sources of credible information regarding sex and contraception in the community [25, 31] • Knowledge of advice regarding safe sex and contraception [34] • Know condoms are preventative against HIV [32] • Know where to access condoms [27, 32, 33] • Knowledge about some available contraceptive methods [30]
	Shame, embarrassment	• Women felt ashamed and shy about accessing condoms [30] • Embarrassment and shame prevents buying or accessing condoms [24] • Shame and stigma prevents attending and asking for contraception in a clinical context [28, 31] • Shame and embarrassment in talking about family planning and contraception with health care providers, parents, sexual partners, in school settings, etc. [18, 26, 33] • Fear of lack of confidentiality and privacy when accessing community health centres [19, 20]. • STI’s not considered shameful in a South-East Northern Territory community, as genital infections are common among men who have undergone the ritual subincision of the penis [35]	• Women reported being able to access and carry condoms without stigmatisation [24]
	Female specific	• Women typically not responsible for condoms [32] • Desire to not use contraception [31]	• Desire to use contraception [31] • Clear fertility intentions and plans for (future) pregnancy [31]
	Male specific	• Men assume condom use is women’s responsibility [23] • Men refuse to wear a condom because they want a baby [5, 33] • Men refuse to wear a condom [24] • Men dislike condoms and prefer sex without [18, 24]	• None reported
	Contraceptive specific	• Men dislike condoms and prefer sex without [18, 24] • Condoms considered protection for men only [32] • Women typically not responsible for condoms [32] • Men assume condom use is women’s responsibility [23] • Condoms impact men’s sexual pleasure (and sometimes women’s) [32] • Condoms primarily considered for STI, not pregnancy, prevention [32] • Negative experiences or unwanted side effects with hormonal contraception, leading to discontinuation or ‘taking a break’ [5, 21, 22, 30] • Dissatisfaction with available methods [31]	• Positive experiences with contraceptive implant [5] • Positive side effects, including lighter periods [5]
Interpersonal	Sexual relationship	• Women suggested that men preferred sex without condoms [24, 30] • Women unable to negotiate condom use [17, 18, 23, 24, 33] • Shame about talking about condom use with partner [33] • Condoms not used in established relationships [24, 27, 32] • Condoms not used because partner’s sexual history is known [34] • Condoms not used because partner does not like them (non-gender specific) [25, 34] • Condoms not used because partner trusted [25, 34] • Couples do not discuss condom use, or sex and reproductive health matters [32, 33, 35] • Men refuse to wear a condom [24] • Partner refused to wear a condom because he wanted a baby, or pressured woman to stop using hormonal contraception for same reason [5, 33] • Do not use contraception because pregnancy is desired [18]	• Couples discuss condom use [33] • Some emphasised importance of condom use and would abstain from sex if one was not available [24] • Using contraception improves sex life by facilitating sex without procreation [21, 22] • Condoms good for casual relationships [24, 27, 32] • Proactively picking up free condoms when sex was anticipated [24]
	Healthcare providers/ educators	• Shame and embarrassment talking about family planning and contraception with healthcare professionals or during sex education in school [18, 26, 33]	• Given advice regarding safe sex and contraception [34] • Positive interactions with health care providers, facilitating contraceptive information provision [31] • Aboriginal nurse aid accompanying women to appointments [28]
	Family/friend relationships	• Shame and embarrassment talking about family planning and contraception with health care providers, parents, sexual partners, in school settings, etc. [18, 26, 33] • Misperceptions, misinformation, and negative experiences of family and friends [31]	• Ability to talk to family and friends about contraception and reproductive health [31] • Mothers supportive of daughters contraceptive use (and even taking them to the clinic for contraception) [5] • Support of extended family (e.g. Aunties) in accessing or using contraception [18] • Mothers report the importance of women not having babies when they are too young [5]
	Community members	• Condom use not sanctioned by community Elders [35] • Negative attitudes towards contraception among some female community members [21, 22, 29, 30]	• Positive attitudes towards contraception among some community members [19–22, 29]
	Context of sex	• Non-consensual sex and sexual assault [17, 24, 33] • Drug and alcohol use among men and women impede ability to practice safe sex [24, 27, 33] • Not using contraception in the heat of the moment as noted by both male and female high school students [18] • Never carry condoms with them [27] • Inability to anticipate sex and therefore do not have condom available [18, 24, 25]	• Using contraception improves sex life by facilitating sex without procreation [21, 22] • Couples discuss condom use [33] • Carrying condoms to be prepared for unplanned sex [24]
Local	Access	• Do not know where to access condoms in community, how much they cost, or know where but not how [32, 33] • Overwhelming information on internet, difficult to identify reliable information [31] • Limited access to contraception in two regional areas of Queensland [18, 31] • Cannot afford cost of IUD [28] • Free condoms sometimes run out [24] • Homelessness exacerbates issue of condom access [24] • Lack of a suitable general practitioner, or other trusted person to provide family planning advice [29] • Lack of culturally appropriate information about contraceptive options, or information provided which assumes a higher level of health literacy than is present, and health care provider does not provide information without judgement [31] • Timing of postpartum contraceptive advice [31] • Fear of lack of confidentiality and privacy when accessing community health centres [19, 20]	• Women reported being able to access condoms without stigmatisation [24] • Able to identify sources of credible information regarding sex and contraception in the community [25, 31] • Given advice regarding safe sex and contraception [34] • Know where to access condoms [27, 32, 33] • Condoms accessible in community, including free condoms [24]
	Cultural appropriateness of services and information	• Lack of culturally appropriate information for men [19, 20] • Lack of culturally appropriate clinical care [19, 20] • Lack of culturally appropriate promotion of contraception and sexual and reproductive health information [29, 30, 35] • Unease in clinical environment [28] • Explanations of contraception and STIs within a western medicine paradigm are not consistent with traditional understandings of the body [30] • Readily available access to condoms at women’s centre not seen as culturally appropriate by women of one remote community [33]	• Aboriginal nurse aid accompanying women to appointments [28]
	Stigma	• Women faced stigmatisation for carrying condoms [18] • Embarrassment and shame prevent buying or accessing condoms [24] • Shame of stigma around sexual assault, as well as condom negotiation, experienced by women of a remote central Australian community [33] • Shame and stigma prevents attending and asking for contraception in a clinical context [28, 31] • Fear of lack of confidentiality and privacy when accessing community health centres [19, 20]	• Women reported being able to access and carry condoms without stigmatisation [24]
Societal	Economic factors	• Homelessness exacerbates issue of condom access [24] • Cannot afford cost of contraception [28]	• Condoms accessible in community, including free condoms [24]
	Cultural norms	• Contraception is taboo [18, 26, 29] • Cultural norms among women from four allied communities in the Northern Territory regarding the female body; women do not expose their pelvic region to strangers, especially men [19, 20] • Cultural norms regarding first pregnancy; women do not use contraception to delay first pregnancy [29, 30] • Within a community in the South-East Northern Territory, cultural norms (e.g. the subincision of penises as a transition to manhood and women not being involved in male health, including penises) limit who is permitted to access condoms, where condoms are able to be distributed, how condoms are perceived, and therefore whether or not they are actually used [35] • STIs are not considered shameful in a South-East Northern Territory community, as genital infections are common among men who have undergone the ritual subincision of the penis [35] • Cultural understandings of the female reproductive body; explanations of contraception and STIs within a western medicine paradigm and not consistent with traditional understandings of the body [30] • Reproduction is highly valued and pregnancy and childrearing acceptable, natural and desirable [30, 31, 35] • ‘Transformative potential of motherhood’ [17] • Cultural and gender constraints prevent women from engaging in condom negotiation [33]	• None reported

#### Intrapersonal level

At an Intrapersonal (i.e. individual) level, the main themes that were elicited from the available data were knowledge, shame/embarrassment, female specific factors, male specific factors and contraceptive specific factors. Knowledge of contraception options, potential benefits, accessibility, and credible information sources on contraception contributed to contraceptive use. Whereas, lack of knowledge or misinformation about available contraceptive options, sexual and reproductive health, and accessibility inhibited use of contraception.

Shame and embarrassment was a predominant theme in many studies, in both remote and urban communities. In a community where women were able to access and carry condoms without stigmatisation, there was no shame attached, therefore facilitating contraceptive use. Fear of being embarrassed or experiencing shame prevented people from accessing contraception or sexual and reproductive health services. Shame in talking about sex and contraception and fear of confidentiality issues also contributed to non-use of contraception or avoidance of health services, education or conversation about sexual health.

Gender specific factors played a role in many communities. Beliefs about which gender was responsible for providing particular contraception, which varied amongst communities, would often contribute to non-use. Males dislike for condoms often contributed to non-use. The desire to have a baby contributed to both males and females not using contraception. Clear plans for family planning and a desire to use contraception as a mode of family planning were factors contributing to women’s use of contraception.

When it came to contraceptive specific factors, contraception was not used when it was seen as reducing sexual pleasure (e.g. condoms), or there was debate as to whether the man or the woman was responsible for providing contraception (e.g. condoms). Negative side effects of hormonal contraception led to its non-use, whereas positive experiences or positive side effects (e.g. lighter menstruation) were seen to influence its use.

#### Interpersonal level

The Interpersonal level of the model consisted of five themes: sexual relationships; healthcare providers/educators; family/friend relationships; community members; and context of sex. Factors within sexual relationships that contributed to the non-use of contraception included lack of conversation about sexual health or negotiation of contraceptive use, desire to have a baby, view that condoms are not needed for established or trusting relationships, and the perception of a partner’s preference to not use contraception. Anticipating an upcoming sexual encounter, facilitating sex without procreation, discussing contraception with a sexual partner, and the view that some contraception, particularly condoms, are good for sex within casual relationships were all factors contributing to contraceptive use.

Relationships and interactions with healthcare providers/educators, family and friends, and community members all influenced the use or non-use of contraception. Within the healthcare providers/educators theme the factors influencing the use of contraception were having had a positive interaction with healthcare providers, being given credible information about sexual health and contraception, and having an Aboriginal nurse aid accompany women to appointments. Feelings of shame and embarrassment with having conversations about sexual health and contraception with healthcare providers or educators often led to non-use. Avoidance of such conversations with some family (e.g. parents) or friends was also seen to prevent use of contraception, whereas talking with other family members (e.g. aunties) or friends who are supportive about the need for contraception may facilitate its use. Mothers viewing that a daughter can be too young to have a baby can also influence the daughter’s use of contraception. The support or lack of support for contraceptive use by community Elders, and the positive or negative views of some community members, can also impact contraception uptake or lack thereof.

Factors related to the context of sex being consensual, enjoyable, safe, anticipated or drug affected also contributed to the non-use or use of contraception. The factors that were reported as influencing non-use were non-consensual sex or sexual assault, drug and alcohol use, unanticipated sexual encounter, and being caught up in the heat of the moment. Being prepared for unanticipated sex by carrying condoms, couples discussing contraceptive use (e.g. condoms), and enjoying sex outside of procreation were all factors contributing to contraceptive use.

#### Local level

Three main themes were used to describe the factors found at the Local level of the ecological model. The first theme was access. Being able to access information and services about sexual health and contraception, as well as being able to access affordable contraception were related to contraceptive use. Where there was a lack of access to: reliable information sources, judgement free and confidential services, trusting healthcare providers for sexual health and contraception, or affordable contraception, non-use of contraception was more likely.

The second theme within the Local level of the model was stigma. When women were able to carry condoms without stigma, this facilitated contraceptive use. Stigma often resulted from women accessing contraception (e.g. condoms) in some communities, sexual assault, and attending clinical appointments or accessing health services for contraception.

The availability of culturally appropriate services and information was the final theme at the Local level. The non-use of contraception was often linked to a lack of culturally appropriate services and information, where particular services or information were not seen as gender appropriate, there was unease within a clinical environment, or the information or services were presented in the Western medicine paradigm, rather than tailored for an individual community [e.g. service developed and implemented in partnership with the local community]. The attendance of an Aboriginal nurse aid at appointments facilitated contraceptive use among Aboriginal women.

#### Societal level

The Societal level, which encompassed all previous levels of the model, contained two themes; economic factors and cultural norms. Economic factors, including homelessness and the inability to afford contraception, exacerbated contraceptive non-use. The availability of affordable or free contraception within the community was related to use of contraception.

Cultural norms varied between the multiple unique communities covered by the included studies. None of the reported norms facilitated contraceptive use. Examples of cultural norms which impacted on non-use of contraception included contraception being viewed as taboo, beliefs about the female body and not exposing the pelvic region to strangers, cultural roles of men and women, rituals involving subincision of the penis to initiate boys into men, and childbirth and pregnancy being valued and desired.

## Discussion

The evidence base exploring the factors influencing contraceptive use and non-use among Aboriginal and Torres Strait Islander peoples is both lacking in quantity and quality. A mere 17 studies published over the last 50 years met the inclusion criteria for this review, and the majority of those did not meet the quality assessment criteria. It is worth noting that the majority of studies conducted in this area, and those rated highest in quality, have been qualitative. However, only two of the included qualitative studies met all quality assessment criteria.

The current evidence base is limited as the included studies did not represent all states and territories of Australia (only studies from Queensland and the Northern Territory were available). In addition, the included studies overwhelmingly focused on women, or women and men together, with only one study focusing on men only. There was a lack of evidence in relation to Torres Strait Islander communities. Half of the included studies focused on young people, and nearly half focused on STIs and risky behaviour. Condoms were the most frequently explored contraceptive method in the literature. Results of this narrative synthesis need to be considered with the lack of quality evidence and specific context of the studies in mind.

This review revealed a multitude of interlinking and complex factors contributing to the use and non-use of contraception among Aboriginal and Torres Strait Islander peoples. The Social Ecological Model assisted in explaining these results in terms of the Intrapersonal, Interpersonal, Local and Societal factors which influence contraceptive use and non-use. To understand one level of factors, it is important to look at it in relation to the other levels of the model. For instance, shame was a prominent theme at the Intrapersonal level (e.g. women feeling shame for carrying condoms), and closely related to stigma (i.e. Local level factor; e.g. stigma of women accessing condoms) resulting from going against cultural norms (i.e. Societal level factor; e.g. gender roles in relation to particular contraception). This impacted communication about contraception within many of the relationships captured by the Interpersonal level.

Some of the factors were highly unique to specific communities, and as such should not be extrapolated to other Aboriginal and Torres Strait Islander communities. In particular, cultural norms were classified as factors influencing non-use in all studies. A number of factors identified in this review are applicable to the broader Australian and international contexts. For example, specific contraceptive characteristics, relationship status and contraceptive and reproductive coercion have all been identified as impacting contraceptive use (or non-use) in various ways for various population groups (for example, Australian women aged 18–23 years) [36–38]. Local level factors were also found to impact contraceptive use and non-use, such as access to culturally appropriate services and affordable contraception. This may be in part attributed to rurality, as privacy concerns in small communities, and access to consistent general practitioner care and other health services have been identified as issues specific to rural and remote locations in Australia [39, 40]. It is unclear if these factors are exacerbated among Aboriginal and Torres Strait Islander communities, particularly given the history of forced contraception upon these communities [3]. Further research exploring the contraceptive experiences of Aboriginal and Torres Strait Islander peoples is warranted among those communities who identify this as a priority.

The strength of this review, using the Social Ecological Model for the narrative synthesis, is that it provides a more holistic, conceptual view of factors which contribute to contraceptive use and non-use within Aboriginal and Torres Strait Islander communities. Although many of the factors identified are applicable to the broader Australian population, the Social Ecological Model allowed for the identification of areas in which positive and collaborative change could facilitate greater access to, and use of contraception (if desired) among specific communities. When working in partnership with communities that have expressed a need for culturally appropriate contraception services and information, it is important to examine the factors within the differing levels of the model that would need to be addressed to tailor services and information in a culturally safe and sensitive manner. Due to the diversity that exists among the hundreds of Aboriginal and Torres Strait Islander communities and their right to self-determination, deciding what is culturally safe and appropriate is not for external researchers or practitioners to define. Rather partners of communities should be guided by the communities themselves. For example, ecological frameworks have been successfully utilised in a number of health promotion programs run in Victoria in collaboration with Aboriginal and Torres Strait Islander health organisations [41], and could potentially be useful in the context of contraceptive practices.

This review has identified only two high quality qualitative studies which explored the factors influencing contraceptive use or non-use among Aboriginal and Torres Strait Islander communities [30, 31]. It was not surprising that the majority of studies included were qualitative in nature, as many research methods are a product of Western concepts that ignore differing views of the world, and may not be appropriate for Aboriginal and Torres Strait Islander peoples [42]. Future research with Aboriginal and Torres Strait Islander communities could help to fill the evidence gap on the existence or non-existence of unmet contraception needs, family planning practices and culturally appropriate services and information. However, any future research or programs implemented in this area should be led by Aboriginal and Torres Strait Islander peoples, with support from researchers and funding bodies, as suggested by national guidelines [42].

### Limitations

Although an examination of gendered differences was planned, the lack of studies reporting gender specific factors influencing contraceptive use or non-use meant this was not possible in most cases. While the search was systematic, it was not exhaustive, and therefore some studies in the area, particularly results reported in grey literature, may not have been identified. Moreover, some of the studies identified were published prior to 2000, with two published in the 1970’s and 1980’s. Availability of and attitudes towards contraception have changed in the intervening years, and findings should also be considered within this context.

## Conclusion

Overall, a multitude of intertwined Intrapersonal, Interpersonal, Local and Societal level factors which influence contraceptive use and non-use were identified in this review. Although there were a number of factors unique to individual Aboriginal and Torres Strait Islander communities (including, for example, cultural norms regarding gender roles and contraceptive use), other factors were not dissimilar to those faced by the broader population, including dislike of particular contraceptive methods, difficulty negotiating contraceptive use with sexual partners, and a lack of knowledge regarding available methods. Understanding factors specific to individual Aboriginal or Torres Strait Islander communities which influence contraceptive use and non-use, as well as factors relevant for the broader Australian population can assist in tailoring contraceptive services for specific communities which have identified a need or desire for such services.

## Data Availability

The datasets used and/or analysed during the current study are available from the corresponding author on reasonable request.

## Abbreviations

LARC: Long-acting reversible contraception
HILDA: The Household Income and Labour Dynamics in Australia
IUD: Intrauterine Device
STI: Sexually transmitted infection
PRISMA: Preferred Reporting Items for Systematic Reviews and Meta-Analyses
CASP: Critical Appraisal Skills Program
QLD: Queensland
NT: Northern Territory
WA: Western Australia
NSW: New South Wales
SA: South Australia

## Footnotes

1 In Australia, many (but not all) contraceptive methods are available to the consumer at a decreased cost. Condoms are widely available at supermarkets and pharmacies. Hormonal methods require a prescription from a healthcare provider.
